# Associations of adult measures of childhood growth with breast cancer: findings from the British Women's Heart and Health Study

**DOI:** 10.1038/sj.bjc.6600972

**Published:** 2003-07-01

**Authors:** D A Lawlor, M Okasha, D Gunnell, G Davey Smith, S Ebrahim

**Affiliations:** 1 Department of Social Medicine, University of Bristol, Canynge Hall, Whiteladies Road, Bristol BS8 2PR, UK

**Keywords:** breast cancer, adult height, adult leg length, adult trunk length, arthropometry, early life risk

## Abstract

Since the two components of adult height – leg length and trunk length – are poorly correlated with each other and appear to be influenced by different early life factors, examining their separate influence on breast cancer may provide additional insights into the mechanisms responsible for the positive association between adult height and breast cancer. In a cross-sectional study of 4286 women aged 60–79 years, in whom there were 170 cases of breast cancer, we found total height, leg length and trunk length were all modestly positively and linearly associated with breast cancer. The magnitudes of the associations of leg and trunk length were similar: fully adjusted odds ratio (95% confidence interval) of breast cancer for a one standard deviation (s.d.) increase in leg length 1.17 (0.98, 1.39) and for a 1 s.d. increase in trunk length 1.19 (0.99, 1.41). Self-reported birth weight (available on 33% of the sample) was positively and linearly associated with breast cancer: fully adjusted odds ratio of breast cancer for a 1 s.d. increase in birth weight 1.30 (0.93, 1.80). These associations were all independent of each other and other potential confounding factors and are likely to reflect different mechanisms by which factors operating prenatally and prepubertally influence breast cancer risk.

Ecological studies of breast cancer have shown that geographic variations in incidence and mortality are associated with variations in population height (Barker *et al*, 1990; Albanes and Taylor, 1990). The majority of cohort studies have found that tall stature is associated with increased cancer risk. The most consistent associations are found for breast cancer, a recent review finding that all but one (Davey Smith *et al*, 2000) of 24 prospective studies showed a positive association with height (Gunnell *et al*, 2001).

The underlying mechanisms for the association between tall stature and breast cancer risk are not understood. The association may be explained by factors that jointly influence stature and breast cancer risk. These include hormones such as insulin-like-growth factor I (IGF-I) (Hankinson *et al*, 1998; Kaaks *et al*, 2002), energy intake in childhood (McCay *et al*, 1939; Hart and Turturro, 1997; Frankel *et al*, 1998), and also intrauterine growth, reflected by birth weight, which has been found to be positively associated with breast cancer risk (Michels *et al*, 1996; Stavola *et al*, 2000; Vatten *et al*, 2002). Since the two components of adult height – leg length and trunk length – are poorly correlated with each other and appear to be influenced by different early life factors (Gunnell, 2002; Wadsworth *et al*, 2002), examining their separate associations with breast cancer may provide additional insights into the mechanisms responsible for the association between height and breast cancer. Three case–control studies have reported inconsistent findings in the associations between components of height and breast cancer (Brinkley *et al*, 1971; Mondina *et al*, 1992; Swanson *et al*, 1996). One prospective cohort study found modest associations between both leg length and trunk length (Albanes *et al*, 1988), whereas a second found an association with leg length only, but this was based on six cases of breast cancer (Gunnell *et al*, 1998). The aim of this study is to investigate the associations of self-reported birth weight and adult anthropometric indicators of childhood growth with breast cancer.

## METHODS

### Participants

The British Women's Heart and Health Study comprises 4286 (60% of those invited to participate) women aged 60–79 years randomly selected from general practitioner lists of 23 British towns. Selection of towns, general practitioners (GPs) and participants was based on the methods used for the British Regional Heart Study of men (Shaper *et al*, 1981). Ethics committee approvals were obtained for the study and consent to access medical records and to flag the women with the National Health Service Cancer Register (NHSCR) were obtained from the participants. Participants completed a questionnaire and attended a local health centre where a research nurse interview, physical examination and blood sampling were undertaken. General practitioner (primary care) medical records were reviewed for each participant and details of diagnoses of cardiovascular disease, diabetes and cancers were extracted by reviewing written general practice records, hospital letters and computerised medical records. Data were collected between April 1999 and March 2001 and full details of methods have been previously reported (Lawlor *et al*, 2002).

### Measurements

Standing and seated heights were measured to the nearest millimetre, without shoes, using a Harpenden Stadiometer. Trunk length was calculated as the seated height minus the height of the stool (407 mm). Leg length was taken as the standing height minus the trunk length. Weight was measured in light clothing without shoes to the nearest 0.1 kg using Soenhle portable scales. Waist circumference was taken as the midpoint between the lower rib and the iliac crest. Hip circumference was taken as the largest circumference below the waist.

Three sources of data were used to determine breast cancer status: (i) women were asked if they had ever been told by a doctor that they had breast cancer and, if so, the date of diagnosis; (ii) diagnoses of breast cancer together with dates were extracted from the general practitioner medical records (including written records, computer records and hospital correspondence); and (iii) all participants were flagged with the National Health Service Central Register (NHSCR), which provided details of cancer registrations. Anyone with a diagnosis of breast cancer from any one of these three sources was considered to be a prevalent case. Flagging of participants with the NHSCR was continuous, and is still ongoing, from the time of the baseline fieldwork (completed in March 2001). We included all cases from the NHSCR that were reported to us by the NHSCR up to November 2002 (the time of the current analyses) and that had been diagnosed prior to the date that the woman attended for baseline study examination. Cases were defined as pre- and postmenopausal based on self-report of age at menopause and the date of first diagnosis of breast cancer obtained from the GP record, cancer register or, if not available from these two sources, self-report (*n*=11). For women who had had an oophorectomy prior to their natural menopause, the date of the oophorectomy was taken as their date of menopause. For women who had had a hysterectomy without oophorectomy prior to their natural menopause, their age of menopause was assumed to be the median of the cohort – 50 years (*n*=11 breast cancer cases), and for women with breast cancer who did not provide details of their age at menopause, their age at menopause was assigned 50 years (*n*=3). The women were asked to report their birth weight in pounds (lb) and ounces (oz) in the self-completed questionnaire; they were not given the option of providing birth weight in prespecified categories. For comparisons with other studies, the self-reported birth weights were converted from lb to kg by multiplying by a factor of 0.4545.

### Statistical analysis

Pearson's correlation coefficients were used to assess associations between total height and components of height. To illustrate the direction and shape of associations, age-adjusted means and prevalences of breast cancer and possible confounding factors were estimated across quarters of height and each component of height. These were estimated using multiple linear and logistic regression models with the age variable centred around the mean age value. Multiple logistic regression was used to assess the associations of the various anthropometric measures with breast cancer prevalence, with adjustment for potential confounding or intermediary variables. In these models, age, age at menarche, age at menopause, weight, waist-to-hip ratio and birth weight were entered as continuous variables. Osteoporosis (yes, no), hysterectomy and/or oopherectomy (yes, no) adult and childhood social class (I, II, III nonmanual, III manual, IV, V) and smoking (never, ex- and current) were entered as dummy variables. Of the 4286 women, 425 could not be assigned an adult social class and 545 could not be assigned to a childhood social class because they did not provide data on occupation. Although the participants were not specifically asked about unemployment, these women are likely to have been married to unemployed men (for those with missing adult data) and had fathers who were unemployed (for those with missing childhood data). This is consistent with the findings that women without these data on social class were more likely to smoke, more likely to be obese, were shorter and were more likely to have prevalent coronary heart disease than cohort members who provided these data (Lawlor *et al*, 2002). In the main analysis, women with missing social class data were allocated to social class V, the most deprived group. A sensitivity analysis was conducted in which these women were excluded from the analysis. These results did not differ substantively from the main analyses and have not been presented in this paper. There were missing data on age at menarche (*n*=405), age at menopause (*n*=280), smoking (*n*=19) and osteoporosis (*n*=476). In all multiple logistic regression models, only those with complete data on all variables (*n*=3554) included in the fully adjusted model were included in each simpler model. In all analyses, robust standard errors taking into account possible nonindependence between women from the same town were used to estimate confidence intervals.

## RESULTS

The prevalence of breast cancer among women who were invited to take part in the study but did not respond was obtained from GP record reviews – the proportion of general practice recorded breast cancer did not differ between responders and non-responders (3.1% (95% confidence interval 2.6, 3.7%) *vs* 2.8% (2.2, 3.5%), *P*=0.7). In total, 170 of the participants had a diagnosis of breast cancer from at least one source, giving an overall prevalence of 4.0% (3.4, 4.6%). The majority (87%) of these cases were identified from at least two sources. The age distributions of women with cancer identified by each source were similar – mean (standard deviation) age of women with breast cancer identified by self-report 68.5 (5.3), identified by medical record review 68.4 (5.3) and identified by cancer register 68.6 (5.3). Of the 170 cases, 39 (22.9%) were premenopausal and 131 (77.1%) were postmenopausal.

While both leg length and trunk length were strongly correlated with total height (age-adjusted Pearson's correlation coefficients 0.80 (95% confidence interval 0.78, 0.82) and 0.76 (0.74, 0.79) respectively), leg length and trunk length were only weakly correlated with each other (*r*=0.24 (0.21, 0.27)). The weak correlation between the two components of height justifies considering their roles in the association between height and disease outcome separately.

Table 1

**Table 1:** Age-adjusted means or prevalences (95% confidence interval) of breast cancer and other characteristics by quartiles of components of height (*n*=4286)

	**Height quartile (range of height in cm)**				
	**1 (115.2–154.7)**	**2 (154.8–158.7)**	**3 (158.8–162.8)**	**4 (162.9–189.9)**	***P*** **trend**
Age (years)	70.3 (69.9, 70.6)	69.1 (68.8, 69.5)	68.1 (68.1, 68.8)	67.3 (67.0, 67.6)	<0.001
Breast cancer (%)	2.8 (1.9, 4.1)	3.9 (2.8, 5.3)	4.8 (3.6, 6.3)	4.8 (3.6, 6.4)	0.03
Age at menarche (years)	13.2 (13.1, 13.3)	13.3 (13.2, 13.4)	13.4 (13.3, 13.5)	13.5 (13.4, 13.6)	<0.001
Age at menopause (years)	47.7 (47.3, 48.1)	48.2 (47.9, 48.6)	48.1 (47.7, 48.4)	48.6 (48.2, 49.0)	0.02
Osteoporosis (%)	10.7 (8.8, 12.9)	9.2 (7.5, 11.3)	8.9 (7.2, 10.9)	6.6 (5.1, 8.4)	0.006
Weight (kg)	65.0 (64.2, 65.8)	68.7 (67.9, 69.5)	70.5 (69.7, 71.2)	74.4 (73.6, 75.2)	<0.001
Waist : hip ratio	0.822	0.821	0.818	0.815	0.02
	(0.818, 0.826)	(0.817, 0.825)	(0.814, 0.822)	(0.811, 0.819)	
Current smoker (%)	13.4 (11.4, 15.6)	10.8 (9.0, 12.8)	9.3 (7.6, 11.2)	10.0 (8.3, 12.1)	<0.001
Adult manual social class (%)	57.6 (54.2, 60.9)	51.6 (48.3, 54.9)	49.9 (46.6, 53.2)	44.4 (41.1, 47.7)	<0.001
Childhood manual social class (%)	82.4 (79.7, 84.9)	77.3 (74.4, 79.9)	76.5 (73.6, 79.1)	69.7 (66.5, 72.7)	<0.001
Birth weight (kg)	2.98 (2.89, 3.07)	3.24 (3.15, 3.30)	3.34 (3.26, 3.42)	3.51 (3.43, 3.59)	<0.001
	
	**Leg length quartile (range of leg length in cm)**				
	**1 (49.8–73.1)**	**2 (73.2–75.7)**	**3 (75.8–78.3)**	**4 (78.4–100.7)**	***P*** **trend**
Age (years)	69.1 (68.8, 69.5)	68.9 (68.6, 69.2)	68.7 (68.4, 69.1)	68.4 (68.1, 68.8)	0.02
Breast cancer (%)	3.3 (2.3, 4.6)	4.1 (3.0, 5.5)	3.3 (2.3, 4.7)	5.5 (4.2, 7.2)	0.13
Age at menarche (years)	13.1 (13.0, 13.2)	13.3 (13.2, 13.4)	13.4 (13.3, 13.5)	13.5 (13.4, 13.6)	<0.001
Age at menopause (years)	48.1 (47.7, 48.5)	48.0 (47.6, 48.3)	47.9 (47.6, 48.3)	48.6 (48.2, 48.9)	0.06
Osteoporosis (%)	8.3 (6.7, 10.3)	8.7 (7.0, 10.7)	9.5 (7.7, 11.6)	8.7 (7.0, 10.7)	0.99
Weight (kg)	67.6 (66.8, 64.8)	68.5 (67.7, 69.3)	70.3 (69.4, 71.0)	72.1 (71.3, 72.9)	<0.001
Waist : hip ratio	0.818	0.821	0.820	0.817	0.96
	(0.813, 0.822)	(0.817, 0.826)	(0.816, 0.825)	(0.812, 0.821)	
Current smoker (%)	11.7 (9.9, 13.9)	12.0 (10.1, 14.2)	8.6 (7.0, 10.5)	11.3 (9.5, 13.5)	0.55
Adult manual social class (%)	58.1 (54.8, 61.4)	51.6 (48.3, 54.9)	47.0 (43.7, 50.3)	46.4 (43.2, 49.7)	<0.001
Childhood manual social class (%)	84.2 (81.6, 86.5)	76.1 (73.1, 78.8)	75.9 (72.9, 78.7)	69.5 (66.4, 72.5)	<0.001
Birth weight (kg)	3.03 (2.94, 3.12)	3.23 (3.14,3.32)	3.56 (3.28, 3.44)	3.47 (3.39, 3.55)	<0.001
	
	**Trunk length quartile (range of trunk length in cm)**				
	**1 (64.9–80.7)**	**2 (80.8–83.0)**	**3 (83.1–85.4)**	**4 (85.5–112.0)**	***P*** **trend**
Age (years)	70.9 (70.6, 71.3)	69.4 (69.0, 69.7)	68.0 (67.7, 68.4)	66.8 (66.5, 67.1)	<0.001
Breast cancer (%)	3.0 (2.1, 4.3)	4.6 (3.5, 6.2)	3.8 (2.7, 5.2)	4.8 (3.6, 6.4)	0.07
Age at menarche (years)	13.3 (13.2, 13.4)	13.3 (13.2, 13.4)	13.3 (13.2, 13.4)	13.4 (13.2, 13.5)	0.33
Age at menopause (years)	47.7 (47.3, 48.1)	48.1 (47.7, 48.5)	48.4 (48.0, 48.7)	48.3 (47.9, 48.7)	0.01
Osteoporosis (%)	13.1 (11.0, 15.5)	8.6 (6.9, 10.6)	7.0 (5.5, 8.8)	6.8 (5.3, 8.7)	<0.001
Weight (kg)	64.1 (63.3, 64.9)	68.2 (67.4, 68.9)	71.2 (70.4, 72.0)	75.2 (74.4, 76.0)	<0.001
Waist : hip ratio	0.827	0.816	0.816	0.816	<0.001
	(0.823, 0.832)	(0.812, 0.820)	(0.812, 0.821)	(0.811, 0.820)	
Current smoker (%)	12.5 (10.6, 14.7)	11.6 (9.8, 13.8)	10.4 (8.7, 12.4)	8.9 (7.2, 10.8)	<0.001
Adult manual social class (%)	54.1 (50.7, 57.4)	51.5 (48.2, 54.8)	49.6 (46.4, 52.8)	48.0 (44.7, 51.3)	0.008
Childhood manual social class (%)	79.1 (76.2, 81.7)	77.5 (74.6, 80.2)	73.7 (70.7, 76.5)	75.5 (72.4, 78.3)	0.004
Birth weight (kg)	3.05 (2.96, 3.15)	3.20 (3.11, 3.29)	3.32 (3.24, 3.41)	3.50 (3.41, 3.58)	<0.001

a For age at menarche based on 3881 participants; age at menopause 4006 participants; osteoporosis 3811 participants; smoking 4267 participants and birth weight 1394 participants.

shows age-adjusted breast cancer prevalence and other characteristics of the participants across quarter of components of height. Breast cancer prevalence shows a graded linear increase with increasing height, leg length and trunk length. Women with shorter legs were younger at menarche. Both shorter trunk length and shorter leg length appear to be associated with younger age at menopause, although the association with trunk length was more consistent across all four quarters. Current smokers and women with osteoporosis had shorter trunk lengths, but neither smoking nor osteoporosis was associated with leg length. Belonging to a manual social class in both adulthood and childhood was associated with reduced lengths of both components of height, but differences were greater for leg length. Birth weight was positively associated with total height, leg length and trunk length.

Table 2

**Table 2:** Odds ratios (95% Confidence intervals) of breast cancer prevalence for a 1 s.d. increase in height, leg length and trunk length

	**Age-adjusted OR (95% CI)**	**Age, lifestyle and reproductive factor adjusted^a^ OR (95% CI)**	**Age, lifestyle, reproductive factor and lifecourse socioeconomic position adjusted^b^ OR (95% CI)**
Height	1.24 (1.04, 1.48)	1.25 (1.04, 1.49)	1.25 (1.05, 1.50)
Leg length	1.15 (0.96, 1.35)	1.17 (0.98, 1.39)	1.17 (0.98, 1.39)
Trunk length	1.21 (1.00, 1.46)	1.19 (0.99, 1.41)	1.19 (0.99, 1.41)

All analyses included in the table are based only on those with complete data for all variables in final model – 3554 with 145 breast cancer cases. OR=odds ratio CI=confidence interval; 1 s.d.=1 standard deviation leg length=4.3 cm, trunk length=3.6 cm, height=6.4 cm.

a Adjusted for age, smoking, weight, waist-to-hip ratio, age at menarche, age at menopause, hysterectomy/oopherectomy, osteoporosis.

b Adjusted for age, smoking, weight, waist-to-hip ratio, age at menarche, age at menopause, hysterectomy/oopherectomy, osteoporosis, adult social class, childhood social class.

shows the odds ratios for breast cancer associated with each component of height with adjustment for potential confounding factors; these analyses are based on 145 cases of breast cancer among 3554 women with complete data on all variables included in the fully adjusted model. There was no difference in the prevalence of breast cancer between women with complete data on all variables included in these analyses and all women in the cohort (4.1 *vs* 4.0%, *P*=0.72). After adjustment for age, smoking, weight, waist-to-hip ratio, age at menarche, age at menopause, osteoporosis, adult social class and childhood social class, a 1 s.d. increase in total height was associated with an increased odds of prevalent breast cancer: 1.25 (1.04, 1.50). Both leg length and trunk length were associated with increased odds of prevalent breast cancer, with the magnitude of the associations being similar for both components of height: fully adjusted odds ratio (95% confidence interval) for 1 s.d. increase in leg length 1.17 (0.98, 1.39) and for 1 s.d. of trunk length 1.19 (0.99, 1.41). Since age at menarche is associated with height and breast cancer, this could potentially act as an important confounder or explanatory factor. However, adjustment for menarcheal age alone did not importantly influence the associations between total height or any of the components of height and breast cancer. With additional mutual adjustment of leg length for trunk length and *vice-versa* both components of height remained independently associated with breast cancer prevalence: fully adjusted (including trunk length) odds ratio for breast cancer prevalence with 1 s.d. increase in leg length 1.14 (0.96, 1.37) and fully adjusted (including leg length) odds ratio for breast cancer prevalence with 1 s.d. increase in trunk length 1.16 (0.96, 1.39). To assess possible selection bias arising through missing data, we estimated age-adjusted odds ratios for each anthropometric variable in the complete data set. The age-adjusted odds ratios for all women (total height: 1.23 (1.05, 1.45); leg length: 1.14 (0.99, 1.34); and trunk length 1.19 (1.01, 1.37)) were similar to those presented in Table 2 for women with complete data included in the multivariable model.

When these associations were examined separately for premeno-pausal and postmenopausal cancers, there were no associations between height or each component of height and pre-menopausal cancer, but total height, leg length and trunk length were all positively associated with postmenopausal breast cancer (Table 3

**Table 3:** Odds ratios (ORs) (95% confidence intervals (CIs)) of breast cancer prevalence for a 1 s.d. increase in height, leg length and trunk length, with separate results for premenopausal and postmenopausal breast cancer

	**Age-adjusted OR (95% CI)**	**Age, lifestyle and reproductive factor adjusted^a^ OR (95% CI)**	**Age, lifestyle, reproductive factor and lifecourse socioeconomic position adjusted^b^ OR (95% CI)**
*Premenopausal cancers*, n=28			
Height	0.95 (0.66, 1.37)	0.98 (0.68, 1.40)	0.99 (0.69, 1.41)
Leg length	0.93 (0.65, 1.33)	0.94 (0.65, 1.34)	0.95 (0.66, 1.46)
Trunk length	0.99 (0.68, 1.42)	1.00 (0.69, 1.42)	1.00 (0.69, 1.43)
	
*Postmenopausal cancers*, n=117			
Height	1.32 (1.09, 1.61)	1.33 (1.09, 1.62)	1.31 (1.07, 1.61)
Leg length	1.19 (0.99, 1.44)	1.23 (1.01, 1.49)	1.21 (1.00, 1.48)
Trunk length	1.29 (1.07, 1.55)	1.26 (1.04, 1.52)	1.23 (1.01, 1.51)

All analyses included in the table are based only on those with complete data for all variables in final model. For premenopausal cancers, *n*=3437 with 28 premenopausal breast cancer cases; for postmenopausal cancers, *n*=3526 with 117 postmenopausal breast cancer cases. Women with premenopausal cancers compared to all women without any cancer. Women with postmenopausal cancers compared to all women without any cancer.

a See Table 2.

b See Table 2.

). In these analyses to avoid contamination, we compared women with premenopausal cancer to women without cancer (i.e. we did not include women with postmenopausal cancers in the analysis) and similarly for the analysis concerning postmenopausal cancers the reference group was women with no evidence of breast cancer.

Of the 4286 participants, 1394 (33%) provided details of their birth weight. There were no differences in prevalent breast cancer between women who provided details of their birth weight and those who did not (3.7% (2.8, 4.9%) *vs* 4.1% (3.5, 5.0%), *P*=0.3). The mean (s.d.) of self-reported birth weights in this cohort was 3.28 (0.80) kg. Self-reported birth weight was weakly positively associated with both leg length and trunk length – age-adjusted Pearson's partial correlation coefficient for the association between birth weight and leg length 0.15 (0.11, 0.19) and between birth weight and trunk length 0.16 (0.11, 0.02). In this subgroup of women with data on birth weights, birth weight was positively associated with breast cancer prevalence – age-adjusted odds ratio (95% confidence interval) of breast cancer for a 1 s.d. (0.80 kg) increase in birth weight was 1.33 (0.96, 1.83). The association was only slightly attenuated in fully adjusted models: 1.30 (0.93, 1.79). Additional adjustment for height had little effect: 1.29 (0.93, 1.79).

Adjustment for birth weight did not significantly alter the associations between total height and components of height with breast cancer (Table 4

**Table 4:** Odds ratios (ORs) (95% confidence intervals (CIs)) of breast cancer prevalence for a 1 s.d. increase in height, leg length, trunk length, leg to trunk length ratio with additional adjustment for birth weight

	**Age-adjusted OR (95% CI)**	**Fully adjusted (but without birth weight)^a^ OR (95% CI)**	**Fully and birth-weight-adjusted^b^ OR (95% CI)**
Height	1.17 (0.85, 1.59)	1.15 (0.83, 1.58)	1.15 (0.83, 1.57)
Leg length	1.13 (0.83, 1.56)	1.14 (0.83, 1.57)	1.14 (0.85, 1.57)
Trunk length	1.23 (0.91, 1.66)	1.20 (0.89, 1.63)	1.16 (0.84, 1.59)

All analyses included in the table are based only on those with complete data for all variables in final model *n*=1250 with 43 breast cancer cases. 1 s.d. 1 standard deviation leg length=4.3 cm, trunk length=3.6 cm, height=6.4 cm.

a Adjusted for age, smoking, weight, waist-to-hip ratio, age at menarche, age at menopause, hysterectomy/oophorectomy, osteoporosis, adult social class, childhood social class.

b Adjusted for all variables included in footnote a and birth weight.

). The fully adjusted (but without birth weight) association between 1 s.d. of height and breast cancer was 1.15 (0.83, 1.58) in this subgroup; addition of birth weight to the model did not change this estimate. Similarly, the odds ratio for the association between leg length and breast cancer was unchanged by adjustment for birth weight. The association between trunk length and breast cancer was slightly attenuated by adjustment for birth weight from 1.20 (0.89, 1.63) to 1.16 (0.84, 1.59), but remained positively associated.

When all analyses were repeated using breast cancer data from each of just one of the three sources (cancer register, general practice medical records, self-report), the results were unchanged. Since socioeconomic position is strongly associated with adult height, we repeated the multivariable analyses with different indicators of socioeconomic position in childhood (car access and bedroom sharing) and adulthood (car access and housing tenure) and found no difference to the multivariable results presented in Tables 2, 3 or 4.

## DISCUSSION

In this study, adult height, leg length, trunk length and birth weight were all modestly and positively associated with breast cancer. The magnitude of the association between total height and breast cancer was similar to that found in a number of prospective cohort studies (Gunnell *et al*, 2001). In one prospective study that looked at the associations of leg and trunk lengths with breast cancer incidence, the fully adjusted odds ratio comparing the top quarter of leg length to the bottom quarter was 1.6 (1.0, 2.6) and that comparing the top quarter of trunk length to the bottom quarter was 1.3 (0.7, 2.2) (Albanes *et al*, 1988). When we analyse our data using a similar approach, the two studies are consistent with each other: odds ratio comparing top to bottom quarter of leg length in our study, 2.0 (1.2, 3.3), and comparing top to bottom quarter of trunk length, 1.8 (1.0, 3.2). We found that total height, leg length and trunk length were associated with postmenopausal breast cancers only. However, the small number of premenopausal cases meant that these results were imprecise; other studies of the height–breast cancer association have found no difference in the associations between height and pre- and postmenopausal cancers (Gunnell *et al*, 2001). The magnitude of the association between birth weight and breast cancer in our study is also similar to that of a prospective study (Stavola *et al*, 2000).

### Study limitations

Our response rate (60%) is moderate but the prevalences of GP recorded cases of breast cancer were similar between responders and nonresponders in our study. Height is significantly affected by socioeconomic position, but the social class distribution in our study sample is similar to that found for the 1991 census (52% manual social class in British Women's Heart and Health Study *vs* 55% older adults in the 1991 census). Further, no indicator of childhood or adulthood social class were found to confound our results. Response bias is, therefore, unlikely to have had an important effect on our results.

Our study is cross-sectional and one of the most important limitations is survivor bias. Breast cancer in the UK is associated with a survival rate of 70% over 5 years (Coleman *et al*, 1999). Our study may, therefore, exclude a number of women with the most aggressive form of breast cancer. This would have an important effect on the results if height and the components of height were associated with breast cancer survival. However, the one prospective study that compared the association between both height and breast cancer incidence and mortality found no difference in the magnitude of the association, indicating no effect of height on survival (Tretli, 1989). Reverse causality must also be considered. Women with breast cancer may become shorter in the trunk because of bone or lung metastases or treatment effects on bone integrity, but this would create an inverse relation (not the observed positive association) with breast cancer. Breast cancer is unlikely to have an effect on leg length, and clearly will not affect birth weight. The associations found in our study are all of a similar magnitude to those found in prospective studies (Stavola *et al*, 2000; Gunnell *et al*, 2001), suggesting that the cross-sectional nature of our study has not significantly biased our results.

We used self-report of birth weight, which may be inaccurate, although self-reported birth weight is strongly correlated with hospital records among middle-aged and older women (Allen *et al*, 2002). Women in our study were born between 1919 and 1940 and the mean self-reported birth weight for these women was 3.28 kg (s.d. 0.80), which is consistent with hospital records of women born between 1923 and 1930 in Hertfordshire, England (3.42 kg, no s.d.) (Fall *et al*, 1995), and also with women in the 1946 British cohort (3.32 kg, s.d. 0.49 – Dr D Kuh, personal communication). Any misclassification bias is likely to have been nondifferential and will therefore have diluted rather than exaggerated the associations presented.

### Explanations for the associations between leg length, trunk length and birth weight with breast cancer

A number of factors may explain the associations between measures of foetal and childhood growth and breast cancer. Genetic factors could explain the association between growth and cancer in two ways. First, genes important in the control of growth may also produce mitogenic proteins. Second, there may be linkage between genes regulating growth and cancer-causing genes. As positive height–cancer associations have been reported in dizygotic twins who were discordant for height, this suggestion is unlikely, although firmer evidence is required from studies of growth–cancer associations in monozygotic twins. Age at menarche is closely related to patterns of growth and final height (Okasha *et al*, 2002). The timing of exposure to the high levels of sex hormones experienced during puberty may be related to breast cancer risk. However, adjustment for age at menarche did not substantially alter the associations between total height or either component of height and breast cancer.

Leg length and trunk length are only weakly correlated with each other and their associations with breast cancer are independent of each other and other potential confounding factors. This suggests that each component of height is likely to be associated with breast cancer via different mechanisms. Table 5

**Table 5:** Possible pathways underlying growth–breast cancer associations (adapted from Okasha *et al*., 2002)

Growth as a biomarker for other exposures that influence breast cancer risk
1. In utero *exposures*
Foetal nutrition is influenced by maternal steroids with greater exposure resulting in greater foetal growth
Exposure to maternal steroids during breast development *in utero* may increase cancer risk
2. *Infections*
Chronic infection may stunt growth – in particular trunk length
Absence of childhood infection may make individuals susceptible to infections, which if acquired in later life are associated with cancer risk and may result in an underdeveloped immune function
3. *Calorie intake*
Lower calorie intake in childhood results in shorter stature and in particular shorter leg length
Lower incidence of cancer in rats fed a calorie-restricted diet
Childhood calorie intake may be related to adult cancer
Calorie intake may also influence growth-promoting hormones (see below)

Growth as a biomarker for biological mediators of risk
4. *Cellularity*
Greater trunk length may reflect a larger number of breast cells
A larger number of cells increases the risk that one will undergo malignant transformation
5. *Growth-promoting hormones*
IGF is related to growth, particularly leg length
IGF is affected by nutritional intake during childhood
IGF is associated with cancer in animal studies although results from epidemiological studies are inconsistent
6. *Oestrogen*
Low lifetime exposure to oestrogen will result in shorter trunk length because of osteoporotic collapse
Later age at menarche will result in greater leg length and total height and may be associated with reduced lifetime exposure to oestrogen
Breast cancer risk is associated with increased exposure to oestrogen

summarises the possible pathways underlying the growth – breast cancer associations, and these are discussed in more detail below with respect to the specific associations identified in this study.

### Leg length

Leg length is an indicator of prepubertal environmental circumstances; the interruption of growth during childhood results in short leg length (Gunnell, 2002). While a number of childhood factors, such as stress, infection and nutrition, may interrupt growth and hence result in short legs, a detailed analysis of the British 1946 birth cohort found that infant feeding was the most important childhood factor associated with leg length (Wadsworth *et al*, 2002). Animal studies have shown that energy restriction leads to growth retardation and decreased risk of cancer (McCay *et al*, 1939; Hankinson *et al*, 1998), and a study in humans found that lower energy intake in childhood was associated with reduced cancer risk in adulthood (Frankel *et al*, 1998). The association between energy restriction in childhood and decreased breast cancer risk in adulthood may be mediated via IGF, which plays a fundamental role in somatic growth. Growth hormone affects cellular growth through the actions of IGFs. Serum IGF levels are also affected by diet (Okasha *et al*, 2002). In children IGF-I levels are positively associated with stature, and during pubertal growth periods IGF-I serum levels can be as high as four times the normal adult concentration. In energy-restricted mice, administration of IGF-I reverses their decreased cancer risk (Petridou *et al*, 2000). However, in epidemiological studies inconsistent findings have been reported for the association between IGF-I and breast cancer (Hankinson *et al*, 1998; Kaaks *et al*, 2002).

### Trunk length

Childhood illnesses lasting longer than 3 months in any year or leading to a hospital admission are associated with shorter trunk length (Wadsworth *et al*, 2002), suggesting that the association between longer trunk length and breast cancer prevalence may reflect reduced childhood infections. A lower number of infections in childhood (resulting in longer trunk length) may lead to increased cancer risk in two ways (Gunnell *et al*, 2001). First, it may leave older children and adults susceptible to infections that, if experienced in later life, carry a greater risk of malignancy. Late childhood infection with Epstein–Barr virus and cytomegalovirus may be implicated in breast cancer aetiology (Richardson, 1997; Touitou *et al*, 2001). Second, it may lead to an underdevelopment of immune function and hence an increase in cancer risk. It has been shown that risk of lymphoma is increased among individuals from small families and that small family size is, in turn, associated with greater stature (Bonelli *et al*, 1990; Davey Smith *et al*, 2000). In the US Nurses Health Study, women who were breastfed as children, and therefore likely to have had fewer childhood infections than those who were not, had a slightly increased risk of breast cancer 1.11 (0.88, 1.39) (Michels *et al*, 2001), supporting the hypothesis that childhood infections might protect against breast cancer risk. We found that women who shared a bedroom as children (and by implication were therefore, more likely to be exposed to childhood infections) were less likely to have breast cancer: age-adjusted odds ratio (95% confidence interval) comparing those who shared a bedroom to those who did not 0.81 (0.59, 1.13), but bedroom sharing did not attenuate the association between trunk length and breast cancer.

Trunk length will also be significantly influenced by lifetime exposure to oestrogen and will be shorter because of osteoporotic collapse in those with low levels of oestrogen exposure. Breast cancer is oestrogen dependent, with risk being lower in those with lower levels of exposure. Thus, the positive association between trunk length and breast cancer may reflect the associations between high levels of lifetime exposure to oestrogen and decreased osteoporosis but increased breast cancer risk.

An alternative explanation for the association between trunk length and breast cancer is that women with larger trunks may have greater breast size. It has been hypothesised that breast size predicts a woman's risk of breast cancer because of increased breast cell numbers, and both chest size (as indicated by bra size) and breast size (as indicated by cup size) have been found to predict breast cancer (Egan *et al*, 1999). Breast density, a marker of cellularity of the breast, is related to breast cancer (Saftlas *et al*, 1991), suggesting that associations between measures of breast size and breast cancer may reflect greater numbers of breast cells leading to increased risk.

### Birth weight

The association between birth weight and breast cancer risk most likely reflects intrauterine exposures to maternal hormones that influence both foetal growth and intrauterine development of the mammary gland. Oestrogen would be one plausible hormone since exposures to high levels of intrauterine oestrogen lead to increased foetal size (Trichopoulos, 1990) and indictors of pregnancy oestrogen concentrations have been shown to be associated with breast cancer risk (Ekbom *et al*, 1992, 1997).

### Implications

The associations between different measures of foetal and childhood growth most likely reflect differing intrauterine and childhood environmental risk factors for breast cancer. These findings highlight the importance of foetal and early life exposures in the aetiology of breast cancer. Further research should concentrate on testing specific hypotheses concerning these associations as outlined in Table 5.
